# Moderate Hypertriglyceridemia Causing Recurrent Pancreatitis: A Case Report and the Literature Review

**DOI:** 10.1155/2018/8714390

**Published:** 2018-09-24

**Authors:** Vijay Gayam, Amrendra Kumar Mandal, Pavani Garlapati, Mazin Khalid, Arshpal Gill, Khalid Mowyad

**Affiliations:** ^1^Department of Medicine and Gastroenterology, Interfaith Medical Center, 1545 Atlantic Avenue, Brooklyn, NY, USA; ^2^Department of Medicine, Wayne State University/Detroit Medical Center, Detroit, MI, USA

## Abstract

Recurrent acute pancreatitis secondary to hypertriglyceridemia (HTG) with levels below 1000 mg/dL has been rarely reported in the literature. HTG is the third most common cause of acute pancreatitis and has been established in the literature as a risk factor when levels are greater than 1000 mg/dL. A 43-year-old patient presented to the hospital with severe epigastric abdominal pain. Initial laboratory investigations were significant for a lipase level of 4143 U/L and a triglyceride level of 600 mg/dL. Computed tomography (CT) of the abdomen showed diffuse enlargement of the pancreas consistent with pancreatitis. A diagnosis of severe acute pancreatitis secondary to high triglycerides was made based on the revised Atlanta classification 2012. The patient was initially managed with intravenous boluses of normal saline followed by continuous insulin infusion. Diabetic Ketoacidosis (DKA) was ruled out due to a past medical history of diabetes. Her clinical course was complicated by acute respiratory distress syndrome requiring intubation and mechanical ventilation. During the course, she improved symptomatically and was extubated. She was started on nasogastric feeding initially and subsequently switched to oral diet as tolerated. After initial management of HTG with insulin infusion, oral gemfibrozil was started for long-term treatment of HTG. Emerging literature implicates HTG as an independent indicator of poor prognosis in acute pancreatitis (AP). Despite the paucity of data, the risk of developing AP must be considered even at triglyceride levels lower than 1000 mg/dL.

## 1. Introduction

Acute pancreatitis is the leading gastrointestinal cause of hospitalization in the United States [1]. The two most common causes of acute pancreatitis are gallstones and alcohol [2].

Hypertriglyceridemia is the third most common cause of acute pancreatitis and accounts for 1 to 4 percent of total cases [3]. It has been established in the literature as a risk factor when levels are higher than 1000 mg/dL [4]. Moderate hypertriglyceridemia is defined as triglyceride (TG) levels between 200 and 999 mg/dL [5]. Although hypertriglyceridemia is an established risk factor for acute pancreatitis, the precise relationship between acute pancreatitis and triglyceride levels remains unclear. In acute pancreatitis, there appears to be a process of triglyceride conversion into toxic free fatty acids (FFA) by pancreatic lipases resulting into lipotoxicity [6, 7]. The severity of acute pancreatitis in patients with HTG is dependent on both the inflammatory response caused by acute pancreatitis and the injury caused by lipotoxicity from triglyceride hydrolysis. We present a patient with recurrent acute pancreatitis secondary to elevated triglyceride levels that remained below 1000 mg/dL. Secondary causes were also considered, as other conditions can elevate triglycerides and can culminate in HTG induced acute pancreatitis [8]. In our patient, a workup of secondary causes of hypertriglyceridemia was unremarkable apart from a past medical history of diabetes.

## 2. Case Presentation

A morbidly obese 43-year-old female with a history of hypertension, dyslipidemia, and diabetes mellitus presented with severe abdominal pain. She had an episode of acute pancreatitis one year ago. She complained of right upper quadrant pain radiating to the back over 6-hour duration alongside six episodes of vomitus. A review of systems was only notable for a headache and dizziness. She reported no family history of dyslipidemia or acute pancreatitis. She denied tobacco, alcohol, or illicit substance use. There was no history of gallstones, appendectomy, new medications, procedures (including ERCP), or any complications related to her diabetes.

Admission vitals revealed afebrile patient with a heart rate of 103/min, respiratory rate of 20/min, BP 116/62 mmHg, and oxygen saturation of 96% on a nasal cannula at 5 liter/min. The patient was alert and orientated but was in moderate distress. The abdomen was obese and soft and with tenderness in the epigastric region. There was no guarding, rigidity, or Murphy's sign. Her body mass index (BMI) was 47.1. Other systemic signs of elevated triglycerides including xanthelasma, corneal arcus, and tendon xanthoma were absent.

Initial laboratory investigations showed an elevated white cell count of 16.9 *μ*/L (4.5 -11 *μ*/L), haemoglobin 12.2 g/dL (12-16 g/dL), platelet count 368000 mm^3^ (130,000-400,000mm^3^), sodium 129 mEq/L (136-144 mEq/L), potassium 3.8 mEq/L (3.5-5mEq/L), anion gap of 2 (8-16), BUN 45 mg/dL ( 7-20 mg/dL), creatinine 0.6 (0.4-1.3), glucose 206 mg/dL (74-117 mg/dL), and serum calcium 7.9 mg/dL (8.5-10.2 mg/dL). Liver function tests showed total bilirubin 0.5 mg/dL (0.1-1.2mg/dL), aspartate aminotransferase 16 IU/L (8-46 IU/L), alanine aminotransferase 11 U/L (7-55 IU/L), total protein 8.5 g/dL (6.1-7.9 g/dL), albumin 4 g/dL (3.5-5.5 g/dL), and PT 11.6 seconds (9.8-13.4 seconds). Initial arterial blood gas (ABG) analysis showed pH 7.5, pCO2 37.5, and pO2 67 at FiO_2_ of 40%.

**Figure 1:**
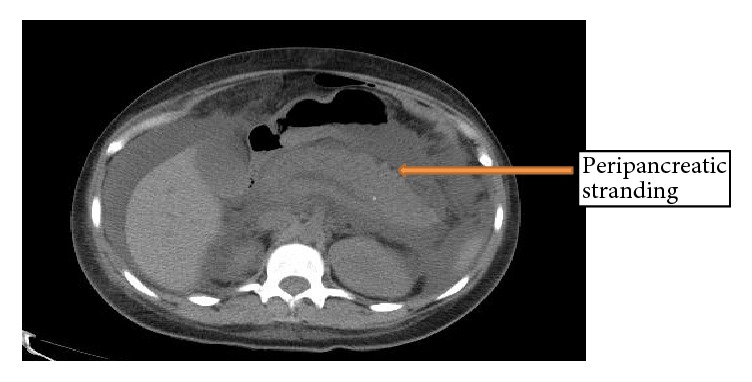
CT scan of the abdomen showing acute pancreatitis (arrow).

The patient had an elevated lipase of 4143 U/L (reference 22-51 U/L), total cholesterol of 694 mg/dL (100-199 mg/dL), and a triglyceride level of 600 mg/dL (reference 0-149 mg/dL). Computed tomography (CT) of the abdomen showed diffuse enlargement of the pancreas consistent with pancreatitis (Figure 1). There was no evidence of gallstones or biliary sludge which was also confirmed by an ultrasound abdomen. Other competing etiologies including alcohol, autoimmune pancreatitis, abdominal trauma, pancreatic divisum, sphincter of Oddi dysfunction (SOD), viral infection, drugs, and toxins were all ruled out, making the most likely diagnosis hypertriglyceridemia-induced AP (HTG AP).

The patient was treated with boluses of intravenous (IV) normal saline and supportive care. On the second day of admission, the patient developed hypotension and had persistent tachycardia. She likely developed acute respiratory distress syndrome (ARDS) from complications of acute pancreatitis. Fluid overload was ruled out with a sonogram of the inferior vena cava (diameter of 1.5 cm), a central venous pressure of 6 cm of H2O, and echocardiography indicating left ventricular ejection fraction of 55% with no left ventricular diastolic dysfunction. The patient was subsequently transferred to the intensive care unit for hypovolemic shock and respiratory distress requiring intubation and mechanical ventilation. As per revised Atlanta classification, she was categorized as severe acute pancreatitis based on Marshall scoring system for organ failure with a score of 3, with a score of 1 from respiratory failure (PaO_2_/FiO_2_ of 240) and two from cardiovascular (systolic BP <90, not fluid responsive). Patient's fasting blood glucose was 288 mg/dL (74-117 mg/dl), and diabetic ketoacidosis (DKA) was ruled out with the absence of serum or urine ketones, no elevated anion gap, and no acidosis. ABG analysis showed pH of 7.5, bicarbonate of 23, and an anion gap of 2.

Following the patients decline into shock, an ABG indicated an acidic pH of 6.99, PCO_2_ 9.4, PO_2_ 134, and HCO_3_ of 5.4. The serum bicarbonate level was low at 15mmol/L and the calculated anion gap was elevated at 22 (normal 8–16). Lactic acid level was 3.8 mmol/L (0.5-1.9 mmol/L).

Repeat abdominal contrast-enhanced CT on the third day showed marked pancreatic and peripancreatic infiltration consistent with AP and no signs of necrosis. Continuous insulin infusion was started for HTG from day 1, lowering her triglyceride levels down to 247 mg/dL over five days as shown in Table 1. She was also started on a liquid diet via nasogastric tube from day 5. After nine days in the ICU, the patient was successfully extubated and was switched to oral diet on day 11, which was gradually advanced as tolerated. She was downgraded to the floor on the same day and discharged on day 17. The patient required nasal oxygen to maintain saturation as she was recovering from ARDS. She was discharged on gemfibrozil 600mg twice daily to prevent further episodes of HTG AP. Patient has been followed upon discharge and remains compliant with gemfibrozil, leading to a controlled triglyceride level of 123 mg/dL and no further episodes of AP.

## 3. Discussion

Acute pancreatitis has several etiologies, but alcohol and gallstones make up the majority of the cases [9]. The exact mechanism of acute pancreatitis (AP) remains unclear and has been hypothesized as an imbalance between proinflammatory and anti-inflammatory cytokines [10]. Hypertriglyceridemia (HTG) is the third most common cause in literature and is classically considered a risk factor with levels higher than 1000mg/dL [4]. Our patient had a triglyceride (TGs) of 597 mg/dL one year ago during her first episode of AP. In the present case, TGs were 600 mg/dL. Our findings were consistent with those of Zhang et al. who observed hypertriglyceridemia-related acute pancreatitis in 102 patients with TG between 500mg/dL and 1000mg/dL. The same cohort of patients also had a high AP recurrence rate of 14.71%. However, the period between recurrent episodes of AP was not established [11].

There is emerging literature implicating HTG as an independent indicator of poor prognosis in AP [12]. Elevated serum TGs in patients with AP are independently and proportionally correlated with persistent organ failure regardless of etiology. However, severe or very severe HTG plus high lipase levels (>3 times the upper limit of normal) are associated with elevated free fatty acid (FFA) levels and can further be complicated by systemic inflammation from acute pancreatitis [12].

A prior meta-analysis concluded that HTG AP is associated with worse outcomes including higher mortality and the higher rates of systemic inflammatory response syndrome (SIRS) as compared to non-HTG AP [13]. Sue et al. studied 2519 patients and observed that patients were more likely to develop persistent organ failure when triglycerides levels were elevated [14]. They also noted even a modest HTG of >200mg/dL could be a statistically significant risk factor for ICU admission. This highlights the need to consider elevated triglycerides and obtain these labs in patients with presumed HG AP to determine etiology and to serve as a prognostic factor as in our patient who required ICU admission.

Management is important in HTG AP due to a high risk of complications. Insulin infusion has been used in literature successfully [15, 16]. However, selected patients with severe HTG AP might benefit from plasmapheresis, but evidence to support its use needs to be validated from various trials [17, 18]. Our patient was started on insulin infusion followed by fibrate to control triglycerides levels and prevent further episodes of HTG AP. Further studies are needed to establish HTG AP in patients with triglyceride levels below 1000mg/dL. Meanwhile, clinicians should be aware of the possibility of developing acute pancreatitis with a triglyceride level lower than 1000 mg/dL. Insulin infusion should be considered in these rapidly reduced triglyceride levels.

## Figures and Tables

**Table 1:** Showing trend of Triglycerides level during the hospital stay.

Lab parameters	Day 1	Day 3	Day 5	Day 11
Triglyceride level	600 mg/dL	393 mg/dL	247 mg/dL	165 mg/dL
